# A peculiar case of syphilis infection: The great imitator is on the rise

**DOI:** 10.1016/j.idcr.2022.e01521

**Published:** 2022-05-18

**Authors:** MJP Eijmael, RG de Bruin, V. Hira, T. Koster

**Affiliations:** a Department of Internal Medicine, Groene Hart Ziekenhuis, Gouda, The Netherlands; b Department of Internal Medicine, Leiden University Medical Center, Leiden, The Netherlands; c Department of Medical Microbiology and Infection Prevention, Groene Hart Ziekenhuis, Gouda, The Netherlands

**Keywords:** Uveitis, Ocular syphilis, Tertiary syphilis

## Abstract

The incidence of syphilis is on the rise worldwide and can pose many diagnostic and therapeutic difficulties for doctors. Given the wide variety of presenting symptoms, syphilis is also known as the great imitator, which in turn frequently leads to a pronounced diagnostic- and therapeutic delay for patients. Here we present a case report of syphilitic uveitis and papillitis accompanied by acute vision loss, a rare presentation of a tertiary syphilis infection that clinically mimicked a giant cell arteritis (GCA) or arteritic anterior ischemic optic neuropathy (AAION). The patient was treated with high-dose intravenous benzyl penicillin after which full vision was restored. By presenting this case, we hope to raise awareness for the increasing incidence of syphilis infections and stress the importance of syphilis testing in patients with otherwise unexplained uveitis.

## Introduction

Over the past several years, the incidence of syphilis has been on the rise worldwide [1], [2], [3]. In the Netherlands, the center for Sexual Health reported an increased incidence of roughly 17% in 2019 as compared to 2018 [4]. Although there has been some attention to this rising incidence, the diagnostic delay and therefore adequate treatment of this infectious disease remains striking, even when patients present with classical symptoms [5], [6], [7].

**Fig. 1:**
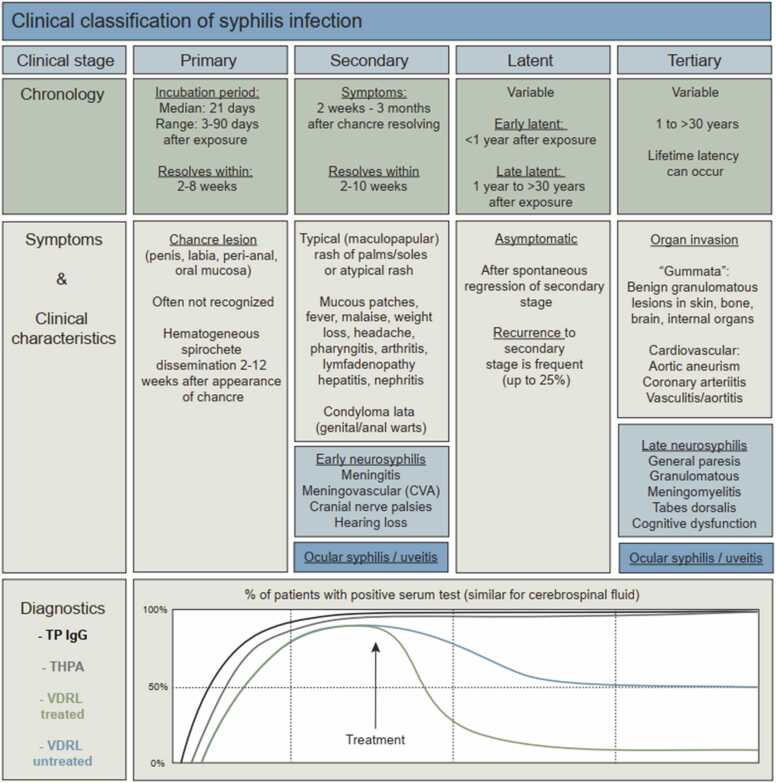
Summary of chronology, symptoms, clinical characteristics and diagnostics corresponding to each clinical stage of syphilis infection.

Syphilis is a usually sexually transmitted disease caused by the spirochete bacterium *Treponema pallidum* [8]. Three stages can be clinically distinguished during the course of infection. Primary syphilis, the earliest stage, can result in overt symptoms after approximately three weeks after inoculation, with a range of 10–90 days (Fig. 1) [9]. It is traditionally characterized by one single, round and painless genital or anal ulcer called a “chancre”, but occasionally more ulcers might be present. The classical ulceration is often not recognized as a sign of syphilis infection as it also spontaneously disappears after two to eight weeks [10]. If left untreated, the infection usually progresses in the secondary stage. This stage can present by a plethora of symptoms such as fever, fatigue, weight loss, a sore throat, patchy hair loss, lymphadenopathy, recurrent genital and/or oral ulcers or a skin rash that can be nonspecific, but classically located at the palms of hand and feet [9]. In this stage, spirochetes can be found throughout the body whereafter spontaneous remission marks the start of the latent stage. This asymptomatic and non-contagious stage has been reported to last for 10–30 years in certain individuals [11], [12]. It can relapse into the secondary stage or can pass into tertiary syphilis, a potentially fatal stage in which the spirochetes can invade diverse organ systems such as the brain, eyes, heart, nerves, liver, bones and joints [9]. Not much is known about the incidence of ocular syphilis. Earlier, a study showed that of the 4232 syphilis patients, 63 (1.5%) were diagnosed with ocular syphilis [13]. On the other hand, around 9% of patients presenting with uveitis were identified to suffer from ocular syphilis [14]. Given the usually protracted disease course due to the diagnostic delay and the large variety of affected organ systems with their respective symptoms, syphilis is often called “the great imitator”, resulting in a marked delay to adequate diagnosis and therefore treatment.

Next we present a rare case with tertiary syphilis that illustrates the diagnostic and therapeutic challenges of a syphilis infection.

## Case presentation

We report a case of a fifty three year old female born in the Dominican Republic, migrated to the Netherlands 33 years prior, with no relevant medical history, who presented to the ophthalmologist with pain and redness around the right eye, initially classified as a scleritis. She was treated with topical steroids and referred to the rheumatologist for further evaluation of possible underlying auto-inflammatory syndromes or infectious diseases. She tested positive for syphilis by positive chemiluminescence immunoassay (CLIA) screening and Treponema pallidum hemagglutination assay (THPA), with a negative rapid plasma antigen (RPR) test, supporting the diagnosis of a preexisting latent or tertiary syphilis infection. She reported no earlier syphilis infection and no prior antibiotics use.

At this time it was unclear whether the syphilis infection was directly related to the reported scleritis of the right eye. Our patient did not report any promiscuous sexual relations and tested negative for other sexually transmitted diseases (STDs) such as *Chlamydia trachomatis*, gonorrhea, hepatitis B and C. Importantly, she also tested negative for HIV, which is often simultaneously present as a co-infection [15]. She was planned to receive intramuscular benzyl penicillin (2.4 million units, administered three times at one week intervals) as a treatment for latent syphilis. Also, her partner tested negative for syphilis, HIV and other STDs at his general practitioner.

Shortly thereafter, she presented with an acute episode of loss of vision in de left eye, upon ophthalmological examination a measured vision of 0.3, with papilledema/papillitis. She also reported a unilateral, left sided headache, with jaw claudication and left sided pain in the scalp, clinically highly suspect for Giant Cell Arteritis (GCA, also known as arteriitis temporalis). Laboratory results however, showed a mildly elevated C-reactive protein (CRP) of 33 mg/l and erythrocyte sedimentation rate (ESR/BSE) of 40 mm/hour and other laboratory findings were normal (Table 1). Lumbar puncture (Table 1) showed no spirochete activity in the liquor cerebrospinalis (negative THPA-test and negative RPR test without signs of inflammation). As a GCA can cause permanent blindness if not treated swiftly [16], she was admitted to our hospital and empirically started on high-dose intravenous methylprednisolone (1000 mg per 24 h) during three days according to national and local guidelines. An atypical presentation of an anterior ischemic optic neuropathy (AION) or non-arteritic anterior optic neuropathy (N-AION) was also considered, but appeared less likely clinically. While using steroids, a concomitantly diagnosed latent tuberculosis infection was treated with isoniazide/rifampicine (600 mg/300 mg once daily for four months).

**Table 1:** Laboratory findings and lumbar puncture.

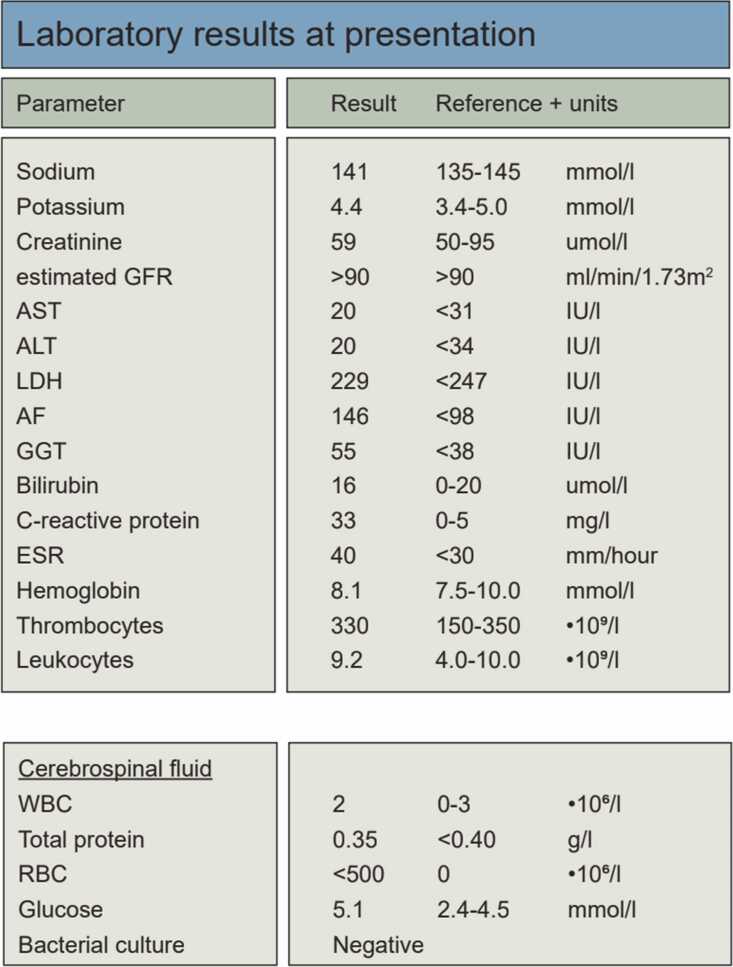

Surprisingly, further evaluation using positron emission tomography (PET-CT) and biopsy of the temporal artery showed no signs of GCA, after which the prednisone was quickly tapered and stopped. Moreover, PET-CT did not show any other signs of inflammatory/infectious activity throughout the body. After multidisciplinary reevaluation, we concluded that the most likely diagnosis is a tertiary stage ocular infection of syphilis, also known as syphilitic uveitis. She was subsequently treated with high-dose intravenous benzyl penicillin (24million units/24 h) during two weeks. A few weeks later, she reported markedly improved vision, with eventually no residual vision loss.

## Discussion

Known as “the great imitator”, we present a rare case of an syphilis infection that involved the eye and optic nerve, clinically mimicking a GCA or A-AION. We illustrate the diagnostic difficulties caused by the pleiotropic clinical presentation of a syphilis infection. Although we primarily treated our patient as a GCA, through thorough multidisciplinary evaluation, we conclude that the ocular findings are consistent with ocular syphilis infection and have treated our patient with high-dose benzyl penicillin with good clinical response. Due to the invasive character of the procedure, no ocular anterior chamber puncture was performed to ultimately prove our diagnosis.

We also illustrate that a negative THPA or VDRL test in the cerebrospinal fluid does not exclude an ocular or optic nerve involvement. Indeed, in a retrospective study focusing on patients with syphilitic uveitis, Bollemeijer et al. [17] showed that the TPHA test was positive in 100% of the serum samples, whereas only 57.9% tested positive in de cerebrospinal fluid. The VDRL-test, which is more or less equivalent to the RPR test we performed, was even less sensitive in this regard, as 81.2% tested positive in serum, versus 38.7% in the cerebrospinal fluid [17].

Here, the striking diagnostic delay is furthermore emphasized as we deduce that our patient probably obtained the syphilis infection from an earlier sexual partner while still living in the Dominican Republic more than 30 years prior, since she only had one other sexual partners who tested negative. Indeed, latent syphilis infections have been reported to last more than 30 years [11], [12]. Moreover, although the vast majority of new syphilis infections are diagnosed in men having sex with men or sex-workers, our case demonstrates that a syphilis infection should always be considered in all patients presenting to the ophthalmologist with unexplained ocular inflammatory symptoms, in particular when uveitis or papillitis is seen. Several previous studies show that all structures in the eye can be affected: A large study by Furtado et al., report posterior uveitis in 76.1% of eyes infected with *Treponema pallidum,* intermediate uveitis and pan-uveitis in 8.4%, anterior uveitis in 6.1%, and isolated scleritis in only 0.9% [18]. Furthermore, this study also showed that papillitis, a sign of posterior uveitis, occurred in 31.1% of affected eyes and loss of vision is frequent [18], which is in accordance with our patients clinical characteristics. Interestingly, in other studies pan-uveitis was most often diagnosed [17], [19], [20] and optic neuritis/papillitis is regarded as a rare phenomenon in immunocompetent patients [21], [22], [23], [24], [25].

In conclusion, by presenting this case we hope to have illustrated the clinically pleiotropic symptoms of a syphilis infection that can result in major diagnostic delay for patients. Furthermore, we hope to have raised more awareness for the increasing incidence of syphilis in general, and in particular the possible ocular manifestations. We therefore stress the critical importance of syphilis testing in patients with otherwise unexplained uveitis.

## Consent

Written informed consent was obtained from the patient for publication of this case report. A copy of the written consent is available for review by the Editor-in-Chief of this journal on request.
